# P029. Migraine, body weight and psychological factors in children and adolescents

**DOI:** 10.1186/1129-2377-16-S1-A153

**Published:** 2015-09-28

**Authors:** Samuela Tarantino, Cristiana De Ranieri, Monica D'Ambrosio, Alessandro Capuano, Roberto Frusciante, Federico Vigevano, Simonetta Gentile, Massimiliano Valeriani

**Affiliations:** Headache Center, Division of Neurology, Pediatrico Bambino Gesù, IRCCS, Rome, Italy; Unit of Clinical Psychology, Ospedale Pediatrico Bambino Gesù, IRCCS, Rome, Italy; Center for Sensory-Motor Interaction, Aalborg University, Aalborg, Denmark

## Background

Several studies have assessed the associations between adult migraine and underweight, pre-obesity or obesity. Prevalence, frequency, and severity of migraine appear to increase in relation to the body mass index, although this evidence is not supported by all the studies examined. The link between body weight and headache has hardly been examined in children. Data on the possible association between the body mass index (BMI) and the psychological profile in migraine children are sparse.

## Objectives

Aims of the present study were: 1) to study the prevalence of pre-obesity and obesity in migraineur children/adolescents; 2) to analyze the possible relationship between frequency and severity of migraine and overweight; 3) to explore the role of anxiety and somatization on BMI in migraine patients.

## Methods

We studied 92 migraineurs (mean age 11.3±2.3 years; 43 M and 49 F). Patients were divided into 2 groups according to headache attack frequency: 1) high frequency (HF) patients, having from weekly to daily episodes, and 2) low frequency (LF) patients, showing ≤ 3 episodes per month. Pain intensity was rated on a 3-level graduated scale (mild, moderate and severe pain). Given the low frequencies, the “moderate” and the “mild” intensity were collapsed into the same category. The psychological profile was assessed by SAFA Anxiety and Somatization scales. BMI was calculated as the weight in kilograms divided by the height in meters squared.

## Results

Among our patients, fifty-seven (62.0%) were classified as “normal weight”, 15.2% were obese and 17.4% pre-obese (both collapsed into the “overweight” group). Due to their low frequencies, “underweight” children/adolescents (5.4%) were eliminated from our subsequent analysis. The weight (“normal weight” or “overweight”) did not correlate with migraine frequency and intensity (respectively: χ^2^) = 0.6853, p = 0.41; χ^2^ = 0.0058; p = 0.94). Compared to normal weight children, overweight patients showed a significant higher score in “Separation anxiety” subscale. In the “overweight” patients, the BMI showed a positive and significant correlation with Anxiety (SAFA-A Total, p = 0.000) and with Somatization (SAFA-S Total, p = 0.000).

## Conclusions

Our results suggest that, in young patients, there is an association between migraine, weight and psychological symptoms. Overweight migraineur patients are more prone to “separation anxiety”. In particular, we can hypothesize that overweight in migraine children may be related to anxiety and somatization symptoms.

Written informed consent to publish was obtained from the patient(s).

